# Making Implementation Science Work for Children and Adolescents Living With HIV

**DOI:** 10.1097/QAI.0000000000001750

**Published:** 2018-07-11

**Authors:** Daniella Mark, Elvin Geng, Susan Vorkoper, Shaffiq Essajee, Kim Bloch, Nicola Willis, Bethany Stewart, Sabrina Bakeera-Kitaka, Nandita Sugandhi, Rachel Sturke, Kechi Achebe, B. Jane Ferguson, Marissa Vicari, Chewe Luo, Nande Putta, Grace John-Stewart, Laura Guay, Angela Mushavi, Imran Muhammad, David A. Ross

**Affiliations:** * Paediatric-Adolescent Treatment Africa (PATA), Cape Town, South Africa;; † Department of Psychology, University of Cape Town, Cape Town, South Africa;; ‡ Division of HIV, Infectious Diseases and Global Medicine, University of California, San Francisco (UCSF), San Francisco, CA;; § Fogarty International Center, National Institutes of Health, Bethesda, MD;; ║ HIV Section, Programme Division, United Nations Children's Fund (UNICEF), New York, NY;; ¶ Africaid, Zvandiri, Harare, Zimbabwe;; # Clinton Health Access Initiative (CHAI), Boston, MA;; ** College of Health Science, Makerere University, Kampala, Uganda;; †† Baylor College of Medicine Children's Foundation-Uganda, Kampala, Uganda;; ‡‡ ICAP, Columbia University, New York, NY;; §§ Save the Children, Westport, CT;; ║║ MARCH Centre, London School of Hygeine & Tropical Medicine, London, United Kingdom;; ¶¶ Africa Health Research Institute, Mtubatuba, South Africa;; ## International AIDS Society, Geneva, Switzerland;; *** Health, Nutrition & HIV Sections, Programme Division, UNICEF, New York, NY;; ††† Departments of Global Health, Medicine, Epidemiology, and Pediatrics, University of Washington, Seattle, WA;; ‡‡‡ Elizabeth Glaser Pediatric AIDS Foundation, Washington, DC;; §§§ School of Public Health and Health Services, George Washington University (GWU), Washington, DC;; ║║║ Ministry of Health and Child Care, AIDS&TB Unit, Harare, Zimbabwe; and; ¶¶¶ Maternal, Newborn, Child and Adolescent Health Department, WHO, Geneva, Switzerland.

**Keywords:** children, adolescents, HIV, implementation science, research

## Abstract

The global HIV response is leaving children and adolescents behind. Because of a paucity of studies on treatment and care models for these age groups, there are gaps in our understanding of how best to implement services to improve their health outcomes. Without this evidence, policymakers are left to extrapolate from adult studies, which may not be appropriate, and can lead to inefficiencies in service delivery, hampered uptake, and ineffective mechanisms to support optimal outcomes. Implementation science research seeks to investigate how interventions known to be efficacious in study settings are, or are not, routinely implemented within real-world programmes. Effective implementation science research must be a collaborative effort between government, funding agencies, investigators, and implementers, each playing a key role. Successful implementation science research in children and adolescents requires clearer policies about age of consent for services and research that conform to ethical standards but allow for rational modifications. Implementation research in these age groups also necessitates age-appropriate consultation and engagement of children, adolescents, and their caregivers. Finally, resource, systems, technology, and training must be prioritized to improve the availability and quality of age-/sex-disaggregated data. Implementation science has a clear role to play in facilitating understanding of how the multiple complex barriers to HIV services for children and adolescents prevent effective interventions from reaching more children and adolescents living with HIV, and is well positioned to redress gaps in the HIV response for these age groups. This is truer now more than ever, with urgent and ambitious 2020 global targets on the horizon and insufficient progress in these age groups to date.

## INTRODUCTION

The global HIV response is leaving children and adolescents behind. Antiretroviral therapy (ART) for children and adolescents is no less efficacious than for adults, yet children and adolescents continue to suffer a disproportionate burden of HIV morbidity and mortality. Children younger than 15 years of age account for 6% of the total people living with HIV globally but 11% of AIDS-related deaths.^1^ Since 2010, AIDS-related deaths among adolescents have decreased by just 5%.^2^ In 2015, AIDS was the fourth most frequent cause of death among girls aged 10–14 years globally, and the fourth most common cause of death among all 10 to 19-year-olds in African low- and middle-income countries, with an estimated cause-specific mortality rate of 17.2 per 100,000 adolescents per year.^3^

Children and adolescents continue to be underserved by services across the HIV treatment cascade.^4^ Of the 2.1 million children younger than 15 years living with HIV, only 43% have access to ART, compared with 54% of adults aged 15 years and older.^5^ Given the rapid disease progression leading to high mortality rates in HIV-positive children,^6^ low early infant diagnosis coverage and linkage to care rates continue to pose significant threats to infant, child and adolescent health, and survival.^7^ Despite improvements in pediatric treatment options, children younger than 15 years continue to have lower viral suppression rates compared with adults aged 15 years and older.^8^ Complicating treatment, children face challenges arising from previous drug exposure during inadequate prevention of mother-to-child transmission, in addition to receiving suboptimal ART formulations themselves which introduce risk of overdosing or underdosing.^9^ A systematic review in resource-limited settings showed that 5–29% of children who initiated ART during the first decade of life were either lost-to-follow-up or deceased by 12 months.^10^ Although HIV testing is the entry point to the HIV treatment, care and support cascade, only 33% of adolescent girls and 20% of adolescent boys in Africa report having ever been tested for HIV.^11^

Treatment adherence and retention of children and adolescents depend on caregivers' ability, willingness, and resources to support their care. Regimens for infants and children are often poorly palatable and require complicated administration techniques, higher pill burden, and frequent dosing changes to accommodate growth, complicating the prescribing and administration of medicine, as well as adherence. Adolescents with HIV face their own challenges arising from their evolving growth and development—they need to transition from pediatric to adult care, seek care independently of a caregiver, balance demands on their time for school and clinic appointments, and build resilience against the stigma and discrimination they will likely experience from their peers, in their schools and in their communities. The operational challenges for both children and adolescents present unique barriers to treatment feasibility and acceptability compared with adults, requiring tailored evidence to inform policy and programming decisions. Without such evidence, policymakers are left to extrapolate from adult studies, which may not be appropriate and can lead to inefficiencies in service delivery, hampered uptake, and ineffective mechanisms to support optimal outcomes in these vulnerable populations.

The importance of implementation science research to investigate, within real-world programmes, how interventions that have been shown to be effective in research settings should be implemented, is being increasingly recognized. This is supported by the recent priority-setting process undertaken by the World Health Organization (WHO) and Collaborative Initiative for Pediatric HIV Education and Research (CIPHER) to develop Global Research Agendas on Children and Adolescents Living with HIV, where implementation came out strongly as an overarching issue, with questions across the 3 thematic areas of testing, treatment, and service delivery for both pediatric and adolescent populations.^12,13^ This article summarizes key elements identified during the development of the research agenda related to conducting implementation science research in children and adolescents living with HIV, examines where we are with implementation science for children and adolescents living with HIV today, and identifies what challenges persist and how they differ for the 2 populations. Potential barriers to conducting implementation science research for children and adolescents and what is needed for success are also discussed.

## USING IMPLEMENTATION SCIENCE WHERE IT IS NEEDED MOST

Although global indicators suggest inferior outcomes in children and adolescents when compared with adults, and that services face particularly severe challenges in providing access for these age groups, systematic reviews^14,15^ reveal a paucity of studies in low- and middle-income countries evaluating effective treatment and care models that address the unique needs of children and adolescents. Although ART efficacy trials in children and adolescents and pediatric cohort data analysis do occur—albeit to a lesser extent than in adults—there are gaps in our understanding of how best to implement routine services for children and adolescents to improve their health outcomes. To date, most implementation studies focus on adult care^16^; however, their results usually do not directly translate to addressing the particular challenges faced by children and adolescents.

Randomized controlled trials are regarded as the “gold standard” in demonstrating efficacy, but exert artificial control over patient, intervention, and setting, none of which are reproducible in routine programs. Although an important part of the investigative toolkit, traditional clinical study design cannot address the translational elements necessary for successful health interventions in real-world settings and as such should not be used to the exclusion of other approaches. Implementing efficacious pediatric and adolescent interventions in routine programs requires services to overcome barriers that also require investigation as part of policy and program planning. Many of these barriers are specific to developmental stage and differ between the 2 age groups. Factors such as caregiver support, regimen complexity, service setting, and role of peers and stigma may impact treatment success differently by age. Unless efficacy trials are supplemented by implementation studies in specific age ranges, our understanding of the true effectiveness of proposed interventions in routine programs will be very limited.

Implementation science research seeks generalizable knowledge as to how interventions that are known to be efficacious in study settings are, should, or should not be implemented within real-world programs.^17^ It aims to improve the quality and effectiveness of health services by identifying, quantifying, and understanding the barriers to, and enablers of, implementation of evidence-based interventions.

Implementation science seeks to understand how both successes and failures happen, and therefore, emphasizes conceptualization and reporting of implementation strategies as well as implementation outcomes.^18,19^ The utility of implementation science findings and its ability to identify interventions that not only work but also can be widely adopted into complex delivery ecosystems, means that it can be of great value in health planning, delivery, and policymaking.

The priorities of implementation science are well suited to investigating the multiple complex barriers to HIV services for children and adolescents. Implementation science has a clear role to play in understanding how these barriers prevent effective interventions from reaching more children and adolescents living with HIV and is well positioned to redress gaps in the HIV response for these age groups. This is truer now more than ever, with urgent and ambitious 2020 global targets on the horizon and insufficient progress in these age groups to date.

## WHAT QUESTIONS CAN IMPLEMENTATION SCIENCE ANSWER FOR PEDIATRIC AND ADOLESCENT HIV TESTING, TREATMENT, AND CARE PROGRAMS?

In 2017, the WHO and Collaborative Initiative for Pediatric HIV Education and Research (CIPHER) led a priority-setting process based on the Child Health and Nutrition Research Initiative (CHNRI) methodology^21^ to identify and score key research questions across HIV testing, treatment, and service delivery based on answerability, impact, implementation, and equity. The exercise resulted in a priority research agenda for child and adolescent HIV testing, treatment, and care aimed at informing global policy and improving patient outcomes.^12,13^

Priority questions were divided into 3 categories: those relating to testing, treatment, and service delivery, and classified according to research type (development, discovery, description, and delivery). Of the 51 total pediatric questions and 61 total adolescent questions, 10 and 13, respectively, were classified as delivery research questions. For children, the highest proportion and total number of delivery questions were included in the testing category (n = 6/16). Similarly, for adolescents, the highest proportion of delivery questions was also in the testing category (n = 5/12), with 3 of those ranking in the top 10 questions in this age group. The highest total number of delivery questions were in the HIV-positive adolescent service delivery category (n = 8/32), the area accounting for over half of the total number of questions related to adolescents. Addressing these delivery-related questions aligns best with an implementation science approach given that priority questions focus on determining effective strategies, interventions, and models to address barriers to treatment and positive health outcomes.

## IMPRACTICAL OR IMPERATIVE?

Despite the clear need for implementation science research for children and adolescents with HIV, the number of published implementation research studies in this field remains limited. This is both an indication and result of insufficient global attention on the pediatric and adolescent HIV epidemic. But in addition, implementation science in these age groups presents challenges to researchers and programmers, which must be overcome.

## BUILDING PARTNERSHIPS ACROSS THE DIVIDE

Service delivery and research tend to be siloed, with different funding streams, implementers, management structures, factors affecting career advancement, and measures of success. Although this is not a challenge specific to the pediatric and adolescent HIV response, it nevertheless creates one of the biggest hurdles to implementation science research in these age groups and must be addressed. Implementation science requires these fractured pieces to come together, through collaboration and alignment of priorities and timelines. Effective implementation science research must be a collaborative effort between government, funding agencies, investigators, and implementers, each playing a key role. Ministries of Health must be provided with the opportunity to contribute to implementation research priorities and to steer the process in country, keeping other stakeholders actively engaged and involved. Evidence emerging from this research should form the basis for policymaking, strategy development, and program implementation. Funding agencies must prioritize implementation science research and provide funding opportunities that align with global and national research priorities.

To undertake implementation research within service delivery settings, investigators are required to ensure rigor within the complexity of prevailing health infrastructure, approval processes and program timelines, cognizant of the existing workloads of health providers, service delivery protocols, health information systems, and targets. Similarly, service implementers are expected to move beyond data compliance to prioritizing the value that evidence can bring to program planning and policy. To achieve this, relationships between investigators and implementers must be characterized by trust and mutual respect. Each has a unique role to play from conception of the research question to dissemination of results, and this must be recognized in defined roles and in the allocation of any external funding for the implementation research. Because implementation science inherently values local relevance and context, implementation science research questions are more effectively conceptualized when informed by collaborations between researchers, implementers, and communities. Investigators may need to support implementers to ensure that program data and the lessons learned in service delivery can be shared in a way that allows for meaningful and useful analysis, interpretation, and application.

Successful implementation research requires implementers to expose suboptimal delivery to researchers, trust them enough to allow them to explore deficiencies within the delivery system and to identify barriers to effective programming. Developing these types of relationships takes effort, usually across long-term collaborations, and is relatively rare. Both parties must be willing to invest in cultivating these relationships and appreciate the distinct constraints under which each is working. Without this commitment, implementers may contend with researchers who fail to understand their context, and researchers may face disinterest and resistance.

Although adapting to a perspective that includes implementation science requires unprecedented interdisciplinary partnership, cooperation, and joint responsibility, research-based practice (or practice-based research) presents a unique opportunity to leverage existing program efforts and resources to improve the quality of services.

## OVERCOMING ETHICAL AND REGULATORY HURDLES

Specific to implementation research in children and adolescents, ethical and regulatory issues pertaining both to age of consent for accessing services, as well as for participating in research, pose serious barriers. Minor assent and parental/guardian consent to research participation are generally required, which may be challenging depending on availability or interest of the parent/guardian to provide consent. In adolescence, the minor may be reluctant to disclose their HIV status and participation in a study to their parent/guardian. Multicountry study design adds further complexity to implementation research because age of consent to services and research both vary across countries. In addition, because children and adolescents are considered vulnerable populations, implementation research in these age groups is subject to greater scrutiny in ethical and regulatory processes.

These facts may result in investigators limiting their research in pediatric and adolescent populations to avoid this complexity or these challenges in obtaining consent, such that enrollment numbers of these countries and age groups are insufficient to draw conclusive age-/sex-specific results. Indeed, the very mechanisms that were instituted to protect the rights and welfare of children and adolescents have created real or perceived barriers to identifying and implementing effective services to support them. To overcome challenges related to consent for service delivery and research with minors, we need to advocate for policies at national level that conform to ethical standards but allow for rational modifications to informed consent procedures to facilitate access to these hard-to-reach age groups, both for the delivery of services and for their inclusion within implementation science research and other studies.

## MEANINGFULLY INVOLVING CHILDREN, ADOLESCENTS, AND CAREGIVERS

Implementation research in children and adolescents necessitates age-appropriate consultation and engagement with children, adolescents, and their caregivers. With increasing age, clients should be partners whose perspectives are valued. Adolescents in particular should share in developing the research agenda and afforded opportunity to identify priorities and reflect on results. With greater equity in the research-participant and provider-patient relationships, the viewpoints and experiences of those engaging with the intervention are leveraged and assimilated into emerging evidence. In the case of infants and younger children, this process requires greater deliberation but should not be abandoned, with the caregiver fully engaged. Diverse methods of facilitating participation across the age spectrum are available^22^ and include visual methods, storytelling, representation of young people on institutional review boards, and young people being involved as peer researchers, for example.

## DISAGGREGATING DATA

Earlier sections have shown that children and adolescents living with HIV have poorer adherence and retention in care, and suffer from worse treatment outcomes than adults. They share many barriers with adults such as stigma and discrimination and financial and time constraints, but they also face unique challenges, such as technical requirements for infant HIV testing, rapid growth and changing formulations/dosages, the level of health worker skill required to respond effectively to their evolving capacities, and the transition between pediatric and adult care.

The rapid physical, cognitive, and social changes that occur across this age group mean that children and adolescents experience a wide variety of individual, family, community, and systems barriers to care. Implementation science research will often primarily depend on routinely collected data. However, despite frequent calls for age- and sex-disaggregated data, these are rarely available from routine programs with sufficient granularity to be useful for studying the particular barriers, and the effectiveness of programmatic changes to overcome these barriers, among children and adolescents. As a result, implementation research across these age groups has often required detailed age-specific studies. These are onerous in requiring large subsamples because the implementation barriers for an infant differ from those of a 18-year-old, for example, and the requirements for an 18-year-old young woman can differ from those of an 18-year-old young man. To improve the availability of age-/sex-disaggregated data and their quality, adequate resource allocation, systems development that makes use of appropriate technology, and training need to be prioritized. Furthermore, as we progress into an era of big data and public accessibility, policymakers should begin to develop an agenda for standardizing indicators, deidentification of data and developing a regional or centralized data hub that can be used by programmers and policymakers to inform high-level decision-making.

## CONCLUSIONS

With super-fast-track targets rapidly approaching,^23^ efforts to expand and improve the identification and treatment of children and adolescents living with HIV must be accelerated. The research agenda must also be fast-tracked to focus on useful, relevant investigations in diverse real-world practice. Without implementation science research to explore the specific barriers to effective treatment for children and adolescents living with HIV, progress is likely to stall. Implementation science cannot be allowed to lag behind in one of the populations that most requires its results.
